# Implementing Educational and Systems-Level Changes to Improve Cancer Screening Rates Among State Employees in Missouri

**DOI:** 10.5888/pcd19.220155

**Published:** 2022-12-01

**Authors:** Misty A. Phillips, Sarah Chavez, Maggie Grotefendt, Xarria Lewis, Melanie Gowdy, Jane A. McElroy, Jean S. Wang, Sandra Hentges

**Affiliations:** 1 Bureau of Cancer and Chronic Disease Prevention, Missouri Department of Health and Senior Services, Jefferson City, Missouri; 2 Siteman Cancer Center, St Louis, Missouri; 3 American Cancer Society, St Louis, Missouri; 4 Gateway to Hope, Maplewood, Missouri; 5 University of Missouri, Columbia, Missouri; 6 Washington University in St Louis, St Louis, Missouri

## Abstract

As of 2022, only 51% of active eligible state employees in Missouri have been screened for colorectal cancer and 67% for breast cancer, despite having state-sponsored health insurance. In fall 2020, the Missouri Department of Health and Senior Services Comprehensive Cancer Program partnered with the Missouri Cancer Consortium to create a strategy to improve cancer screening rates among state employees. The project was designed to include 3 phases: 1) a colorectal cancer education phase, 2) an expanded education phase that included additional cancers, and 3) a proposed intervention phase that will include screening events. In the first phase, in 2020, colorectal cancer educational materials were sent to all state employees. In the second phase, in 2022, educational resources were expanded to include additional cancers and screening tools. In both initiatives, educational materials and information on current screening recommendations were distributed to approximately 40,000 state employees. A database of screening rates was developed to monitor screening rates and challenge state employees to complete screenings. Evidence-informed interventions were implemented with a focus on health equity. We used a regional approach to identify geographic areas with the greatest need. These efforts will support the next phase of the project, which involves planning breast and colorectal cancer screening events. Policy changes will be encouraged to remove systems-level barriers that discourage employees from being screened for cancer. Recommended tools and strategies can be adopted by similar organizations with complex, multitier employee structures.

**Summary**

**What is already known on this topic?**

Missouri state employees have lower rates of cancer screening compared with statewide averages, despite having access to state-sponsored health insurance.

**What is added by this report?**

Our screening improvement program used a strength-based approach to identify existing resources and leverage partnerships to promote collaboration among internal and external partners. We implemented a health education campaign that used print materials, email, videos, and digital media and developed a sustainable and scalable evidence-informed health communications campaign aimed at increasing cancer screening rates among state employees, all of whom have access to health insurance.

**What are the implications for public health practice?**

Results of these efforts have broad implications for public health practice, providing a roadmap for designing and implementing expanded employer-based cancer screening initiatives.

## Background

The Missouri Comprehensive Cancer Control Program (MCCCP) of the Missouri Department of Health and Senior Services (DHSS) is funded by the Centers for Disease Control and Prevention and provides leadership for the coordination of the state’s comprehensive cancer prevention and control efforts (1). To achieve its goals, MCCCP works closely with key partners across the state through participation in the Missouri Cancer Consortium, which is made up of community members, academic partners, health care providers, and other organizations (2). Both MCCCP and the Missouri Cancer Consortium share the overarching goals of preventing cancer and reducing cancer mortality through system-wide advancements in risk reduction, access to early detection methods, improved treatment options, and increased survivorship. Building on a previous work plan, MCCCP collaborated with the Missouri Cancer Consortium to develop a new work plan of goals and strategies for cancer screening interventions among state employees. The goals of this new MCCCP work plan, which also reflect the overarching goals of the Centers for Disease Control and Prevention, are to reduce cancer risk, decrease cancer incidence and mortality, reduce cancer disparities (eg, racial and ethnic, urban vs rural), and improve quality of life among survivors in Missouri. The MCCCP plan has 5 key strategies: 1) enhance the quality and completeness of data submitted to the National Program of Cancer Registries and improve the use and dissemination of data in Missouri; 2) use surveillance systems and population-based surveys to assess the state’s cancer burden and inform cancer control programs; 3) support partnerships for cancer control and prevention; 4) implement evidence-based screening interventions; and 5) monitor and evaluate cancer control programs (3).

### Cancer control programs in the workplace

The collaboration between MCCCP and the Missouri Cancer Consortium was established, in part, in response to a 2016 study that analyzed the extent to which national comprehensive cancer control programs implemented cancer prevention interventions in the workplace (4). A comparison of current workplace screening programs found that tobacco control was the most common intervention, followed by screening for colorectal cancer and breast cancer (4). Workplace interventions were largely focused on education on tobacco cessation (71.9%) or health-related policies on smoke-free work environments (81.3%) (4). Strategies targeting health benefits of interventions were less common (37.5%), but they included sending reminders to employees who were not up to date on screening or providing a health insurance incentive for employees who participate in a health risk assessment and education session (4). Strategies to provide environmental support were focused on reducing access barriers to cancer screening by providing on-site mobile mammography and colorectal cancer screening via fecal immunochemical test (FIT) or fecal occult blood test (FOBT). However, these strategies were reported in the workplans of only 34.4% of comprehensive cancer control programs (4). The study illustrates the need for such programs to include a wider range of interventions to address modifiable cancer risk factors and increase recommended screenings (4). Rates of colorectal cancer screening were lower than rates set forth in national objectives in all comprehensive cancer control programs, suggesting a need for increased program-driven workplace interventions directed toward adults aged 45 to 75 years (4).

### Baseline assessment of cancer screening prevalence among Missouri state employees

Missouri has approximately 40,700 full-time employees working in 24 state agencies, representing 0.007% of the total state population. State employees are a heterogenous population, with a median salary of $41,750, age groups spanning 3 generations, residence in both rural and urban regions, diverse racial and ethnic backgrounds, and a wide range of education and socioeconomic levels. All state employees have access to a state-sponsored health insurance plan (5). Because the state workforce is so diverse, our interventions were designed to promote health equity and overcome any barriers related to social determinants of health (6).

In 2020, MCCCP conducted a needs assessment to compare statewide rates of colorectal and breast cancer screening, screening rates among state employees, and screening rate objectives set forth in Healthy People 2020 (70.5% for colorectal cancer and 81.1% for breast cancer), which have since been updated in Healthy People 2030 (7). Healthy People 2030 objective C-07 is to increase the proportion of adults screened for colorectal cancer to 74.4% (8). At the time of the 2020 assessment only 51.0% of eligible state employees had been screened for colorectal cancer through use of either stool testing or colonoscopy. By comparison, in 2018, 65.2% of US adults aged 50 to 75 years received a colorectal cancer screening based on the most recent guidelines (8), and the 2020 Behavioral Risk Factor Surveillance System survey estimated that 74.7% of people aged 50 years or older in Missouri had ever had colorectal screening (9).

Healthy People 2030 Objective C-05 is to increase the proportion of women in the US who receive a mammogram, with a goal of 80.5% (10). Only 67% of eligible female state employees aged 50 to 74 in Missouri had a mammogram in the previous 2 years. In 2019, an estimated 76.4% of US women aged 50 to 74 years had a mammogram in the previous 2 years in accordance with the most recent guidelines (10). In Missouri, 76.7% of women aged 50 to 74 years had a mammogram in the past 2 years, according to the 2020 Behavioral Risk Factor Surveillance System survey (11). Thus, for both colorectal cancer and breast cancer screening, screening rates among Missouri state employees were lower than state and national rates and lower than Healthy People 2020 and 2030 goals.

## Implementation of the 3-Phase Screening Improvement Project

Implementation of the Screening Improvement Project (SIP) began in fall 2020, with an expected completion date of July 2024. The project team selected a strengths-based approach, defined as a process of capitalizing on assets, strengths, and available resources. Thus, the SIP team began by identifying the resources, strengths, and capabilities of MCCCP to leverage existing partnerships through the Missouri Cancer Consortium. This approach allowed the SIP team to build an evidence-informed intervention based on existing team strengths, including an accessible population, an existing infrastructure for communicating health information to state employees, and collaborative partnerships between state programs and MCCCP. The goal of this project is to increase state employee screening rates by tailoring existing educational materials to a diverse audience of state employees with access to state-sponsored insurance. During early project planning meetings with project partners, the key objective of the SIP was defined as changing the behavior of state employees by encouraging them to develop preventive care-seeking behaviors, such as participating in cancer screenings. Expected outcomes include increases in screening rates, knowledge of cancer prevention behaviors, knowledge of how to access care, and awareness of the importance of cancer screenings; increased work productivity; and decreased time off caused by illness. All of this will result in savings in health care costs, improved health behaviors, and, most importantly, decreased mortality.

The SIP project was designed to include 3 distinct phases directed toward specific populations. The first phase focused on convening partners, disseminating readily available tools and resources on colorectal cancer prevention and screening to state employees, and monitoring overall screening rates over time. The second phase included expanded distribution of education materials to state employees and the development of statewide media campaigns to educate all Missouri residents on screening. The third phase will focus on implementation of evidence-based interventions among state employees and evaluation of these screening events.

## Phase I: Convening Partners and Disseminating Tools and Resources

### Assembling the Screening Improvement Project team

SIP partners were assembled from the Missouri Cancer Consortium, which has multiple workgroups consisting of key partners from around the state, as well as DHSS MCCCP representatives. The partners for this project are the American Cancer Society, DHSS Show Me Healthy Women, DHSS Tobacco Prevention and Control, DHSS Wellness Program, Gateway to Hope, the Masonic Cancer Alliance, the Missouri Cancer Consortium Breast Cancer Workgroup, the Missouri Colorectal Cancer Roundtable, the Missouri Department of Mental Health, the University of Missouri–Columbia School of Medicine, the Siteman Cancer Center, and the Washington University School of Medicine.

The SIP team comprises a diverse group of individuals with a wide range of experience and expertise in cancer control and evidence-based interventions. The team includes 2 master’s-level social workers with experience in strengths-based approach theory. A gastroenterologist joined the SIP team to provide clinical oversight for the interventions, based on her extensive experience with colorectal cancer screening; she is also the chairperson of the Missouri Colorectal Cancer Roundtable. The SIP team also includes a professor who works as an epidemiologist, specializing in cancer prevention, rural health, and health promotion in underrepresented populations, including sexual and gender minorities; a senior scientist to support SIP’s goals to reduce potential disparities in cancer screening and education in the diverse state employee population; a chief executive officer with a unique understanding and perspective on multisector approaches to complex social challenges, such as health care access; and a cancer partnership expert with experience in clinical quality control and improvement. The team’s combined experience is wide ranging and allows for a strong collaboration and partnership for the implementation of SIP’s various phases.

### Identifying a strategy to reach a diverse audience of state employees

The assembled SIP team began by identifying existing assets and expertise that could support the proposed employee screening efforts. This approach builds on the capacity of the existing environment, promotes systems-level change, and encourages preventive care-seeking behavior among state employees. This multitiered intervention was critical to addressing multilevel barriers that could affect preventive care-seeking behaviors, including individual behaviors and resource alignment. By mobilizing available resources and assets of the health landscape, the employer, and the collaborative community partners, MCCCP and the Missouri Cancer Consortium sought to strategically engage in systems-level change to modify the built environment. Identifying and removing barriers for individuals would increase the likelihood of state employees’ potential for successful navigation of systems and use of existing cancer screening resources.

### Dissemination of tools and resources on colorectal cancer to state employees

In fall 2020, colorectal cancer screening rate among state employees enrolled in state-sponsored health insurance plans was 16%. The direct mail brochure featured a selection of photographs that reflected our goal of promoting health equity by representing the diverse population of state employees. These brochures included facts about colorectal cancer, how survival rates are improved if the cancer is caught early, an overview of the screening process, recommendations to lower risk, how to find an in-network physician, and sources of additional information. The materials also encouraged employees to ask their physician about being screened earlier on the basis of additional risk factors, such as family history of colorectal cancer.

The SIP program distributed an email to state supervisors to encourage support of a systems-level policy change. Specifically, we asked supervisors to consider approving employee requests to use their 1 hour per month allocated time for health-related activities, such as participating in a cancer screening event. A 2018 study of the Centers for Disease Control and Prevention–funded Colorectal Cancer Control Program reported higher screening rates in primary care clinics (eg, federally qualified health centers, community health centers) with a champion (a clinical person who assumes a leadership role in evidence-based interventions for CRC screening), further illustrating the importance of developing strong messaging across multiple state divisions and identifying champions to encourage screening at each site (12). To monitor the potential impact of SIP, the SIP team collected data on overall employee cancer screening rates from the state-sponsored health insurance provider. This data collection allowed SIP to track selected locations throughout the state and overall screening rates without having access to individual employees’ personal health information.

## Phase 2: Distribution of Educational Materials, Multimedia Campaigns for All Missouri Residents, Expanded Print Materials

### Expanded distribution of cancer education and screening information

In fall 2021, the prevalence of colorectal cancer screening among state employees enrolled in state-sponsored health insurance plans (50.1%) was 15.1 percentage points less than the prevalence among Missouri population overall (65.2%) (8). Therefore, SIP expanded efforts to disseminate educational materials that described different types of cancer screenings (breast, lung, prostate, cervical, and colorectal) in an electronic format to all state employees. A “Get Your Tests” flyer described guidelines for cancers that have screening tests and explained what each screening entails. The state-sponsored health insurance provider sent an email to all current state employees; the email introduced the educational pieces and referred recipients via an embedded link to a web-based education landing page (healthy.mo.gov/cancer) where they could find additional information on all types of cancers. Attention was given to selecting images of people for the educational material who represented various ages and racial and ethnic backgrounds.

### Multimedia campaigns for all Missouri residents

In April 2022, two multimedia campaigns were developed for all Missouri residents. One focused on cancer prevention and screening (www.youtube.com/watch?v=YcdFuqTM03Q) and one focused on cancer survivorship (www.youtube.com/watch?v=BypYX20kwVI). Both campaigns were run via television advertisements, Facebook, and Instagram to reach adults aged 25 years or older. We based our decision to use television advertisements on the rationale that advertisements viewed on the largest screen in the home (television) are more likely to reach multiple people in a single household simultaneously. The campaign also included static image messages to be shared on social media, such as an infographic on colorectal cancer (Figure). Both strategies focused on providing education on the importance of cancer prevention through risk reduction and encouraging cancer screenings for all people in Missouri.

**Figure:**
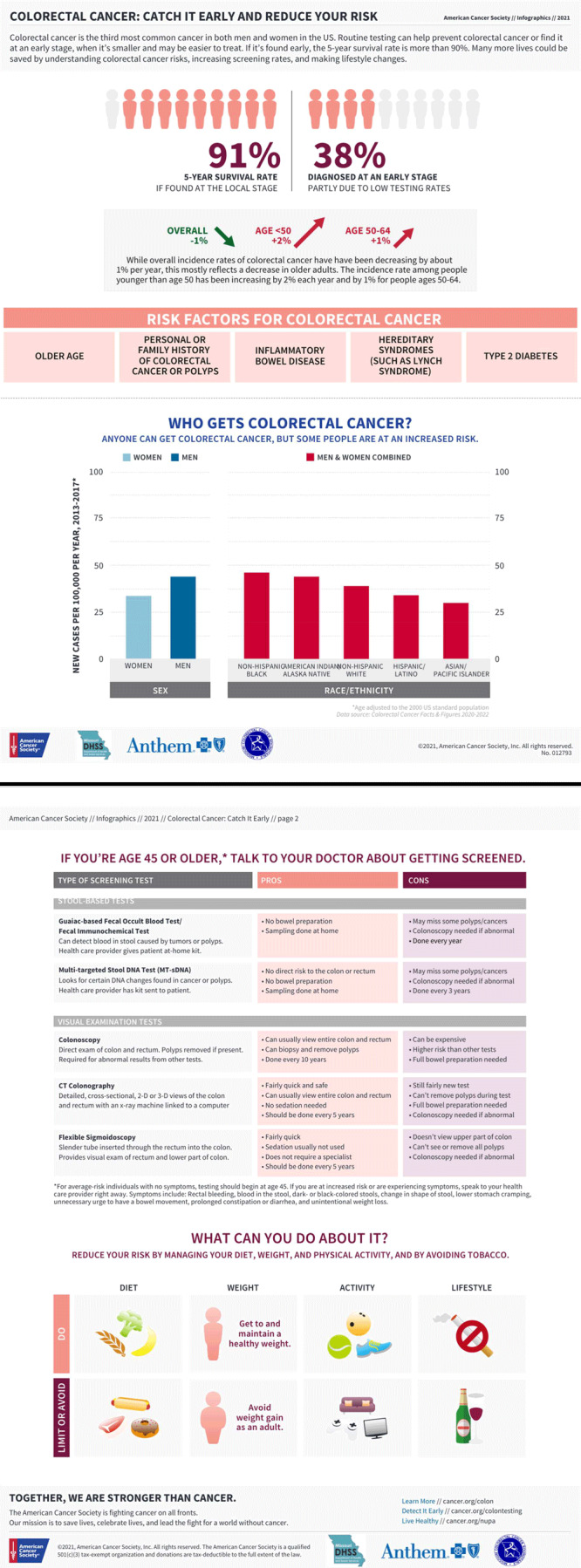
An infographic on colorectal cancer and colorectal cancer screening developed by the Screening Improvement Project in Missouri in 2021. The infographic was designed to increase knowledge and awareness of colorectal cancer and improve colorectal cancer screening rates among Missouri state employees and the general population in the state. Reproduced with permission from the American Cancer Society.

The SIP team solicited recommendations for people to be featured in the media campaigns. As part of the process of selecting people to be featured, the team reviewed answers to a series of questions the cancer survivor provided about their cancer experience. The featured cancer survivors represented various cultural backgrounds, cancers, sexes, ages, and economic statuses. The prevention campaign included three 60-second videos airing as television commercials in May 2022, with more than 5.2 million household impressions. The survivorship campaign included four 90-second videos airing via television and social media across Missouri in June 2022, with the goal of providing support and education related to screening and survivorship. The survivorship campaign reached nearly 1.4 million households, with approximately 15,000 engagements (ie, likes, clicks, comments, and shares).

### Ongoing expansion of print materials for cancer education and screening information (in progress)

In addition to the original cancer education materials, new materials are being developed for lung, colorectal, breast, and cervical cancer. A general cancer screening guide will also be available to print and display in the facilities of 24 state agencies, on bulletin boards throughout buildings and breakrooms frequented by employees. Because some employees may not have reliable access to email or the internet, this phase also seeks to provide accessible resources to state employees not reached through digital media. The development of expanded print materials began in April 2022, with an expected completion by end of summer 2022. By providing both print and electronic versions, we hope to ensure that all employees have access to these materials.

Through implementing these project activities, the SIP team identified several key recommendations to share with other CCCPs (or any employer) seeking to increase cancer screening rates among employees (Table).

**Table:** Project to Improve Cancer Screening Rates Among State Employees in Missouri: Lessons Learned and Recommendations for Implementation

Item	Lessons learned and recommendations for implementation
Print and electronic materials	Using existing evidence-based screening health communications materials developed by the Centers for Disease Control and Prevention allowed for parts of the project to move quickly by only needing to add partner logos.
	Using an electronic platform for parts of the project made some of the materials available quickly and also made it easier to refer employees to additional electronic resources when requested.
	Developing a website landing page for resources allowed for convenient access to a substantial amount of information in a centralized location.
Videos	Developing a script and a schedule provided much-needed guidance to the interviewees, allowing the process to move along quickly and efficiently on a tight timeline.
	Leveraging partners for videos allowed us to capitalize on existing relationships, so we were able to quickly identify experts in the field as well as cancer survivors.
	Meeting interviewees at the marketing agency provided a personal connection with Screening Improvement Project team members and also allowed for edits to be made immediately on-site rather than calling interviewees back for re-shoots.

## Phase 3: Future Directions: Implementation of Evidence-Based Interventions for Screening Events

The strengths-based approach used throughout this project capitalized on resources and capabilities of MCCCP partnerships by building on current evidence-informed interventions and tailoring them to state employees. At least 2 pre-event educational webinars will be advertised to state employees via email announcements in late fall 2022. These webinars will provide information about breast and colorectal cancer and prevention and what to expect when being screened or if receiving an abnormal test result and time for a question-and-answer session. Two physicians will participate on the expert panel, ready to field general medical questions. 

The first on-site event, scheduled for December 12, 2022, will take place in a centralized location in the state capitol, Jefferson City. Fifteen state agency buildings with 3,185 employees are located within a 5-mile radius of the event site. The Ellis Fischel Cancer Center mammography van, which can accommodate 15 appointments per day, will be on site. Approximately 300 stool FIT kits for home-based colorectal cancer screening will be provided by BJC Healthcare/Siteman Cancer Center. Employees who attend the event will complete a brief survey to determine eligibility for screening, and if they are eligible, they will be given a stool FIT kit to take home to complete. Each FIT kit will include a postage-paid addressed envelope for the patient to easily return their stool sample to the laboratory. Test results will be kept confidential, and the referring physician will review results with the patient. Employees with positive test results will be referred for follow-up colonoscopy evaluation. In addition to state employees, other household members on the same health insurance plan can take advantage of the FIT testing opportunity. Others (ie, not state employees or their family/household members) who attend the event and ask about receiving a mammogram or colonoscopy will be provided information about resources. SIP has set a goal of distributing educational pieces to at least 300 attendees at this first pilot event. This event model will be adjusted and replicated at additional events throughout the state in 2023.

Because state employees have diverse demographic characteristics, the SIP team has engaged with the MCCCP Health Equity Workgroup to discuss strategies for planning these events in other parts of the state to reach a diverse population. Our initial selection of evidence-based interventions includes patient reminders and small media (12). Addressing additional barriers will also be considered as we reach out to increase cancer screening behavior in phase 3. For example, in one study, many patients in a safety-net health care setting indicated that their health care provider had not recommended screening or cited concerns about cost and logistic challenges, such as transportation and time (13). Employee reminders will be distributed before the screening events to increase attendance and reduce the number of no-shows.

Evaluation of each of these events — both process evaluation and outcome evaluation — will be completed. The SIP team will meet approximately 2 weeks after each event for a debriefing on such aspects as the pre-event webinars, use of the mammography van, distribution of FIT kits, and volunteers’ feedback. For the quantitative evaluation, we will assess the number of FIT kits returned within 1 month of the event, the proportion of mammogram appointments completed, the number of FIT kits distributed, the number of non–state employees asking for breast or colorectal cancer screening referrals, the approximate number of attendees, and the number of volunteers needed versus available. On the basis of this feedback, we will adjust the logistics of the next event to maximize screening opportunities.

## Summary

Even among state employees with health insurance coverage for routine cancer screening, compliance with cancer screening recommendations fall far short of Healthy People 2030 goals. Collectively, our 3-phase state employee screening project seeks to build on existing resources, strengthen partnerships among organizations and institutions involved in reducing the burden of cancer, and promote preventive care-seeking behavior among state employees. The first 2 phases increased exposure to information about the importance of cancer prevention and screening among state employees and the overall population in Missouri. The capstone events in phase 3, to begin in late 2022, will provide breast and colorectal cancer screening opportunities for state employees. The ultimate success of this 3-phase initiative will be determined by whether cancer screening rates of state employees increase over time. With a state agency providing leadership and a role model, other workplaces have the opportunity to adopt this process with the overarching goal of better health and less cancer burden among Missourians. 
